# Shear–Flexural Performance of Steel Fiber-Reinforced Concrete Composite Beams: Experimental Investigation and Modeling

**DOI:** 10.3390/ma18184322

**Published:** 2025-09-15

**Authors:** Qing Zhi, Zihui Xu, Weimin Chen, Huaxin Zhang, Sha Liu, Zhijun Yuan

**Affiliations:** 1 School of Infrastructure Engineering, Nanchang University, Nanchang 330031, China; zhiqingjr@163.com (Q.Z.); chenweimin@ncu.edu.cn (W.C.); 18379289477@163.com (H.Z.);; 2 School of Astronautics, Northwestern Polytechnical University, Xi’an 710072, China; xuzihui2015@163.com

**Keywords:** composite beam, SFRC, joint, flexural bearing capacity, shear strength, strut-and-tie model

## Abstract

Steel fiber-reinforced concrete (SFRC) exhibits superior tensile and flexural strengths, crack resistance, compressive toughness, and ductility. These characteristics make SFRC attractive for precast beam joints, shear-critical regions without stirrups, and retrofitted overlays, thereby enabling composite members. However, the shear and flexural responses of such members often differ from monolithically cast elements. To clarify these effects, nine composite specimens and one cast-in-place control were tested under four-point bending. Key parameters, including load-bearing capacity, failure evolution, and failure modes, were documented, together with load–deformation behavior, reinforcement strains, and concrete deformations. Results showed that horizontal joints reduced shear resistance and altered crack propagation compared to monolithic beams. Incorporating 1.0% hooked-end steel fibers improved both shear and flexural performance. SFRC above the joint was more effective for shear, while SFRC in both zones improved flexure. The fully SFRC specimen without stirrups achieved 63% higher shear capacity than its NC counterpart, with ductility rising from 2.2 to 3.1. A 1.0% fiber dosage provided shear resistance equivalent to D8@200 stirrups, confirming the potential of SFRC to reduce transverse reinforcement. Analytical models, including a fiber beam–column element and strut-and-tie approach, showed reasonable agreement with experiments.

## 1. Introduction

Steel fiber-reinforced concrete (SFRC) is a fiber-reinforced cementitious composite that has gained significant attention over the past two decades due to its enhanced mechanical properties and crack resistance characteristics [1,2,3]. SFRC exhibits superior compressive toughness, tensile strength, flexural capacity, impact resistance, and fatigue performance compared to conventional concrete [4,5]. Randomly dispersed steel fibers are embedded within the cement matrix, forming a mechanical bridge across microcracks, particularly in the interfacial transition zone between the cement paste and coarse aggregates—areas typically prone to cracking under load [6,7,8,9]. This bridging mechanism substantially improves the post-cracking behavior and toughness of concrete. A comprehensive overview of the tensile characteristics and bond behavior of SFRC was recently presented by Mujalli et al. [10], which highlighted the role of fiber bridging in improving post-cracking toughness and bond capacity.

A number of studies have investigated the mechanical performance of SFRC. Song [11] examined the compressive and splitting tensile strength, modulus of rupture, and toughness index of high-strength SFRC with varying fiber contents (0.5–2.0%). Yoo [12] performed both quasi-static and impact tests on SFRC beams with different compressive strengths (49, 90, and 180 MPa), revealing improvements in flexural strength, deflection capacity, and energy absorption with increasing fiber volume. Wu [13] explored the rheological and mechanical properties of ultra-high-performance concrete (UHPC) incorporating steel fibers of various shapes. Xu [14] studied the effects of aggregate type and steel fiber content on the mechanical performance of high-performance concrete under ambient curing conditions. Gao [15] evaluated the contribution of steel fibers in calculating the flexural capacity of SFRC beams.

It is widely recognized that the addition of steel fibers enhances the shear and flexural performance of reinforced concrete elements. This enhancement is particularly beneficial in critical structural components, such as beams, shear walls, and joints, especially in precast elements subjected to complex stress states [16,17,18]. For instance, Zhao [19] investigated the cyclic behavior of three-story SFRC coupled shear walls, reporting excellent ductility and energy dissipation capacity. Ma [20] conducted experimental tests on five precast beam–column joints with lap-spliced reinforcement, demonstrating that both fiber content and the stirrup ratio significantly improved the damage tolerance and post-peak performance of the joints. Moreover, the use of steel fibers as partial or full substitutes for transverse reinforcement can alleviate reinforcement congestion in heavily reinforced regions, thus improving constructability and reducing labor and material costs. Recent experimental studies have also highlighted the cyclic performance of SFRC beams. Chalioris et al. [21] investigated slender SFRC beams under reversed cyclic loading and demonstrated that steel fibers substantially enhanced crack control, energy dissipation, and post-cracking ductility. These findings further confirm the role of fibers in improving both flexural and shear behavior.

**Figure 1:**
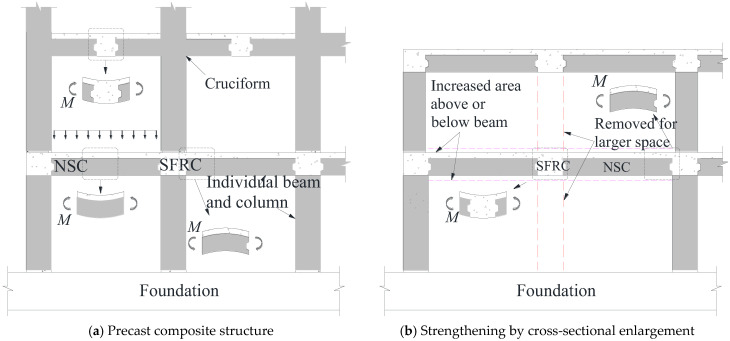
Configurations of composite beams incorporating SFRC.

Figure 1 illustrates typical applications of SFRC in composite beam systems. In one configuration [22], SFRC overlays are applied to precast or existing beams, where under gravity loads, the central portion of the SFRC layer undergoes compression while the ends experience tension. Another application involves strengthening existing precast beams by increasing their cross-sectional area—SFRC is added either on the tension or compression face to enhance overall flexural or shear performance.

Despite these advantages, the practical implementation of SFRC in load-bearing structural members remains limited, primarily due to the lack of reliable mechanical models, particularly for members failing in shear [23]. The shear resistance mechanisms in SFRC beams include contributions from the concrete compression zone, dowel action of longitudinal reinforcement, steel fibers bridging the cracks, and aggregate interlock along critical shear crack paths [24,25,26,27,28]. However, quantifying the relative contribution of each mechanism remains challenging due to the influence of numerous variables, such as the shear span-to-depth ratio, concrete compressive strength, stirrup configuration, aggregate properties, reinforcement details, fiber content, and beam geometry [29,30,31,32,33].

Additionally, in precast or retrofitted structures, construction joints—whether vertical or horizontal—introduce potential discontinuities in stiffness and strength, which may adversely affect structural performance relative to cast-in-place systems [34,35]. Poor bond strength at joint interfaces and abrupt changes in stiffness due to mechanical connectors (e.g., grouted sleeves or lap-spliced bars) further complicate the behavior of such systems. To ensure that the strength, stiffness, and ductility of precast components match those of their monolithic counterparts, high-performance materials, such as SFRC, are increasingly employed at the joint interfaces to enhance local and global structural integrity.

This study focuses on the experimental investigation of SFRC composite beams incorporating horizontal and vertical joints. A series of beams with varying configurations was tested under four-point bending to examine the effects of SFRC location and thickness on flexural and shear behavior. Load-carrying capacity, failure patterns, local deformation, and load–deformation responses were thoroughly recorded. Accordingly, the objectives of this research are to (i) investigate the flexural and shear–flexural behavior of composite beams with and without SFRC, (ii) evaluate the influence of horizontal and vertical joints on structural performance, and (iii) benchmark the experimental results against analytical models, including the ACI sectional method, fiber beam–column element, and strut-and-tie model. These combined experimental and analytical efforts provide a basis for improved understanding of shear–flexural interaction in SFRC members and contribute to the development of more reliable design methodologies.

## 2. Experimental Program

### 2.1. Specimen Design

The specimens were designed with reference to the provisions of ACI 318 to provide a transparent analytical basis for the experimental program. In the initial design stage, the contribution of steel fibers and the presence of horizontal or vertical joints were not considered, and the members were proportioned as conventional reinforced concrete beams. This approach ensured that the reinforcement layout satisfied the minimum code requirements and provided a consistent benchmark for evaluating the subsequent effects of fibers and construction joints.

For the four-point bending configuration, the maximum shear at each support is *V* = *P*/2, and the mid-span moment is *M* = *PL*/4. The nominal flexural strength was evaluated using the following sectional formula for reinforced concrete beams:

(1) $$\;M_{n}=A_{s}f_{y}\left(d-\frac{a}{2}\right),\;\;a=\frac{A_{s}f_{y}}{0.85f_{c}^{\prime }b}$$

where $A_{s}$ is the area of tensile reinforcement, $f_{y}$ is the reinforcement yield strength, $f_{c}^{\prime }$ is the compressive strength of concrete, b is the section width, and d is the effective depth.

For shear, the transverse reinforcement satisfied the minimum stirrup ratio of ACI 318, ensuring constructability and confinement, while the spacing was selected so that shear resistance would not be excessively high and shear-critical responses could still develop. Detailed shear design procedures and model-based predictions are presented later in Section 4.

**Figure 2:**
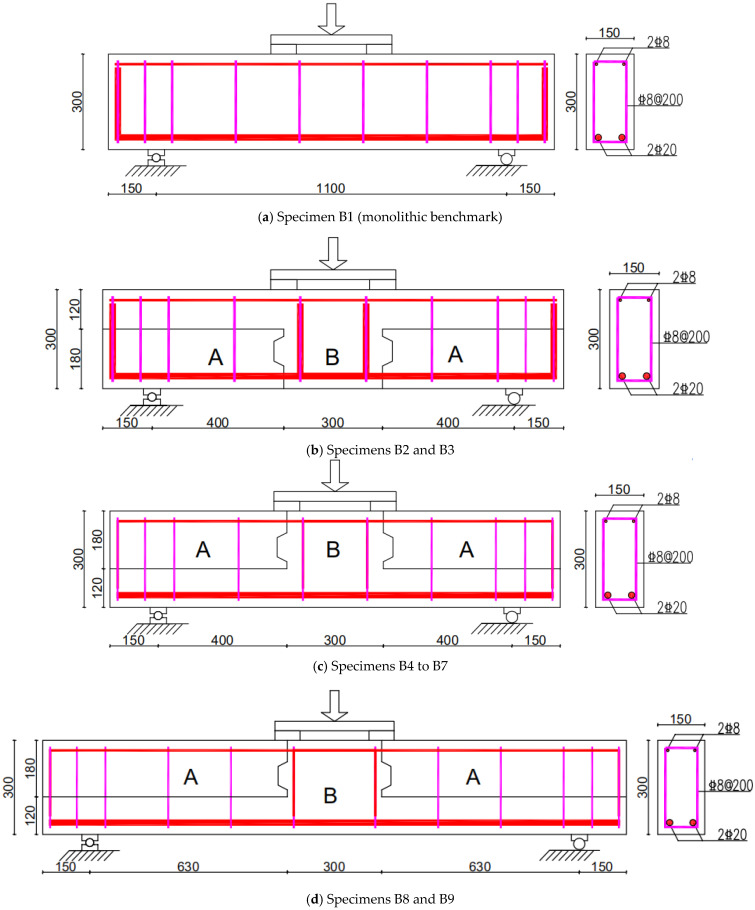
Reinforcement layout and size of the monolithic and composite beams.

Based on these calculations, the short-span beams (≈400 mm, *a*/*d* ≈ 1.5) had a nominal flexural strength of about 52.6 kN, corresponding to a shear demand of $V_{m}$ ≈ 131.5 kN. The nominal shear capacity was $V_{n}$ ≈ 84.0 kN, indicating that shear failure was expected to govern. For the long-span beams (a ≈ 630 mm), the same flexural strength gave a reduced shear demand of $V_{m}$ ≈ 83.5 kN, which was very close to the nominal shear capacity of $V_{n}$ ≈ 84.0 kN, placing these specimens near the shear–flexure transition. The reinforcement layout and specimen dimensions are shown in Figure 2. This design arrangement ensured compliance with code requirements while enabling the intended investigation of shear–flexural interaction and the influence of SFRC placement and construction joints across different shear-span regimes.

A total of nine beams were fabricated, comprising eight composite specimens and one monolithic cast-in-place benchmark for comparison. Each composite beam consisted of precast segments at the two shear–flexural ends and a post-cast segment in the middle zone encompassing the joint region. The vertical interfaces between precast and post-cast segments were formed using shear keys to enhance interfacial bonding.

Seven of the specimens had a total length of 1400 mm, while the remaining two measured 1860 mm. All specimens had identical cross-sectional dimensions of 150 mm × 300 mm. The shear span-to-depth (a/d) ratios adopted in the test matrix were 1.5, 2.1, and 2.4. The reinforcement layout and geometric details of the specimens are presented in Figure 2, and the complete test matrix is summarized in Table 1.

The precast and post-cast segments of the composite beams are referred to as Part A and Part B, respectively. The concrete used in each part was either normal-strength concrete or steel fiber-reinforced concrete (SFRC), depending on the configuration. Notably, two of the specimens were constructed without stirrups in the shear–flexural regions. In structural applications, such as frame systems, the upper and lower regions of composite beams may experience either tension or compression due to lateral seismic loads or complex force interactions. Therefore, in specimens B2 and B3, the precast Part A—located in the tension zone—is placed at the bottom (Figure 2b). Conversely, in specimens B5 to B7, Part A is placed at the top and functions as the compression zone (Figure 2c).

### 2.2. Materials of Specimens

The concrete used was C30-grade, with a specified 28-day compressive strength of 30 MPa, based on standard 150 mm cube tests. Concrete for Parts A and B was cast at different times. During each casting operation, three cube specimens (150 mm × 150 mm × 150 mm) were prepared for both normal concrete and SFRC. The average compressive strengths of each batch are provided in Table 2. Here, the subscripts “n” and “s” refer to normal concrete and SFRC, respectively, while “A” and “B” denote the casting parts.

**Figure 3:**
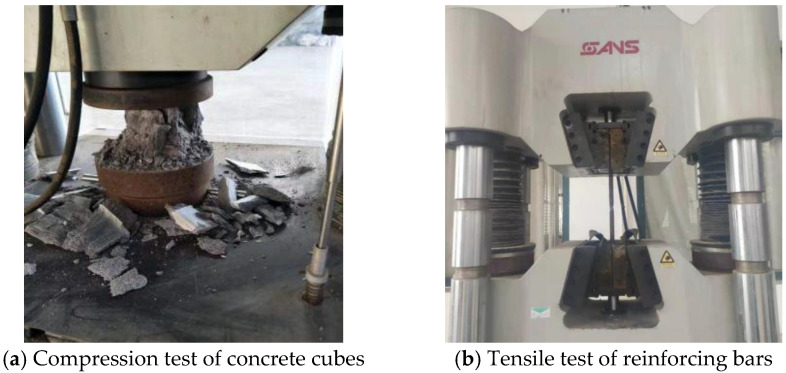
Material tests for strength verification.

The mixed proportions of normal concrete and SFRC with a fiber volume fraction ($V_{f}$) of 1.0% are listed in Table 3. This dosage was chosen as a balance between workability and mechanical enhancement, and is in line with previous studies on SFRC beams. The steel fibers had hooked ends, a length-to-diameter ratio of 30, and a tensile strength of 600 MPa. The longitudinal reinforcement and stirrups were HRB400 ribbed steel bars, with diameters of 20 mm and 8 mm, respectively. The mechanical properties of the concrete and steel bars were verified through material tests, as illustrated in Figure 3 and summarized in Table 3 and Table 4.

### 2.3. Fabrication Process

**Figure 4:**
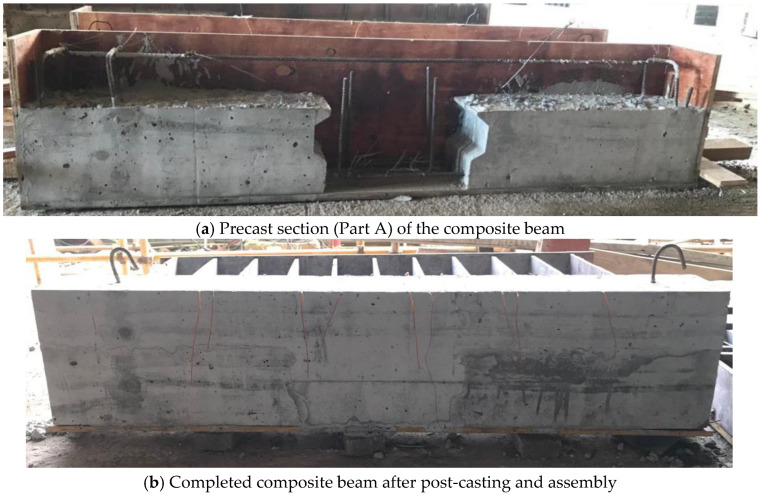
Fabrication procedure for composite specimens.

All beam specimens were fabricated on-site following a consistent procedure. The process involved assembling the formwork, installing steel reinforcement, affixing strain gauges, and then casting and curing the concrete, as shown in Figure 4. Wooden formwork was used for the beam bodies, while steel formwork was employed at the vertical shear key interfaces. To improve the bonding performance between precast and post-cast segments, the horizontal joint interfaces were roughened with a chiseled texture approximately 40 mm wide and 30 mm deep before casting Part B. Following the casting of both sections, the specimens were subjected to curing for more than one month.

### 2.4. Test Setup and Loading Process

**Figure 5:**
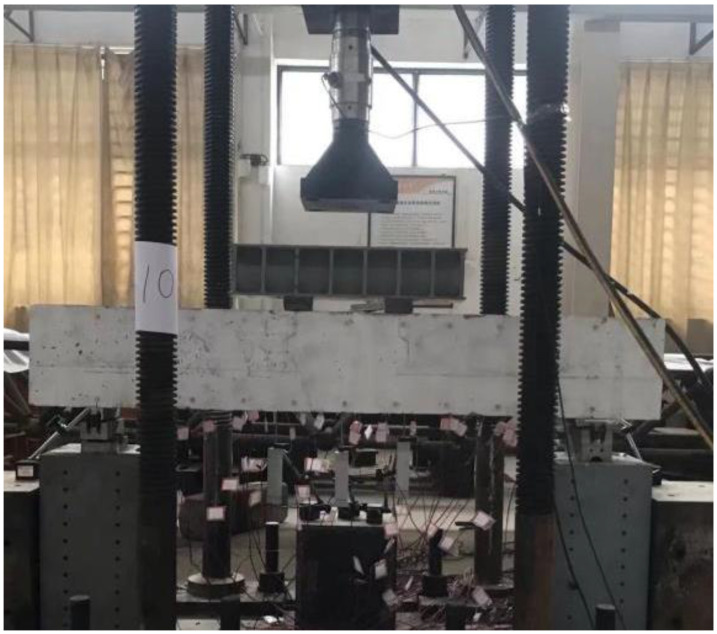
Experimental test setup.

The test setup is illustrated in Figure 5. A four-point bending configuration was adopted for all specimens. The loading protocol employed a multi-stage load-control scheme, with increments of 10 kN applied at a rate of 0.1 kN/s. At each stage, loading was paused once deflection stabilized to observe cracking and record strain data.

**Figure 6:**
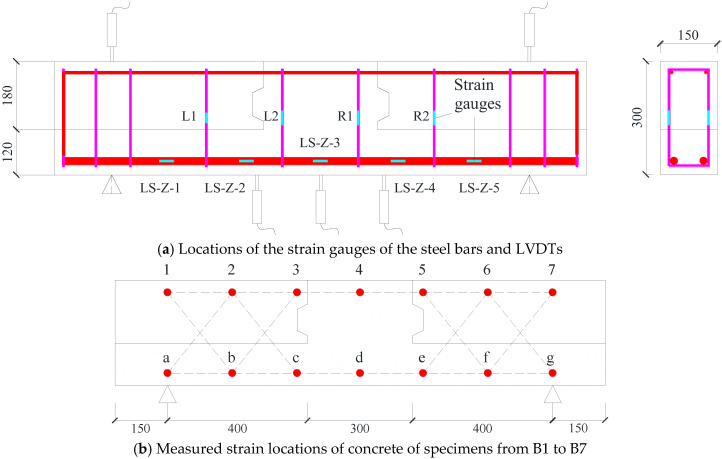

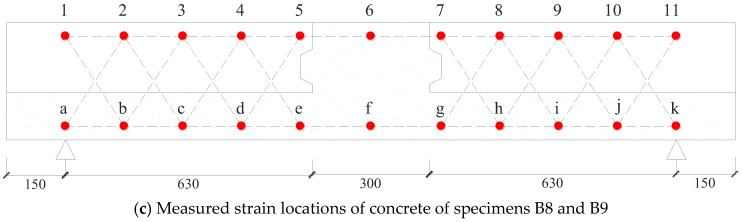
Locations of LVDTs and strain gauges of steel bars and concrete.

Strain gauges were affixed to the longitudinal reinforcement and stirrups to monitor internal stresses during loading. The locations of these gauges are shown in Figure 6a. Concrete surface strains were also monitored at selected regions, as illustrated in Figure 6b,c. Five linear variable differential transformers (LVDTs, Beijing, China) were installed beneath each beam to track mid-span and support displacements throughout the test.

## 3. Test Results and Discussion

### 3.1. Failure Modes

**Figure 7:**
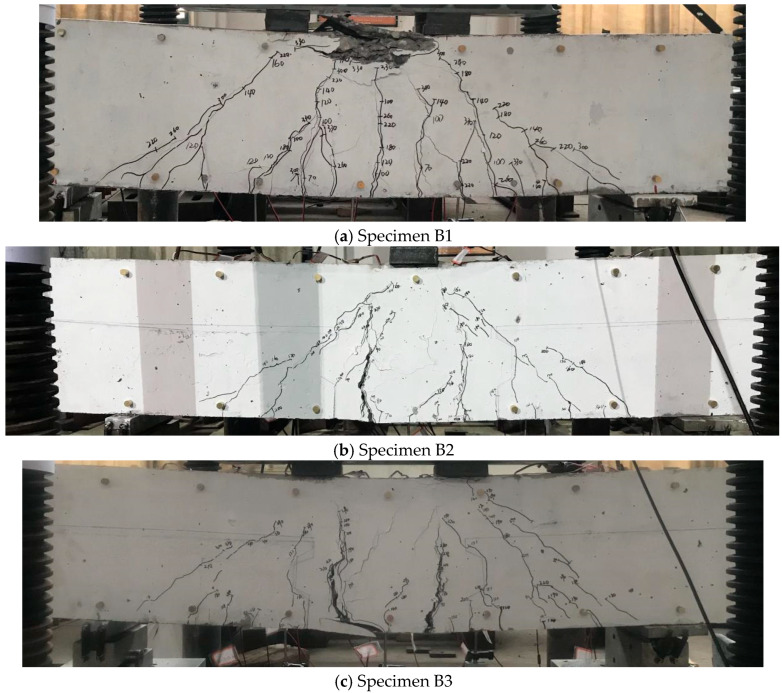

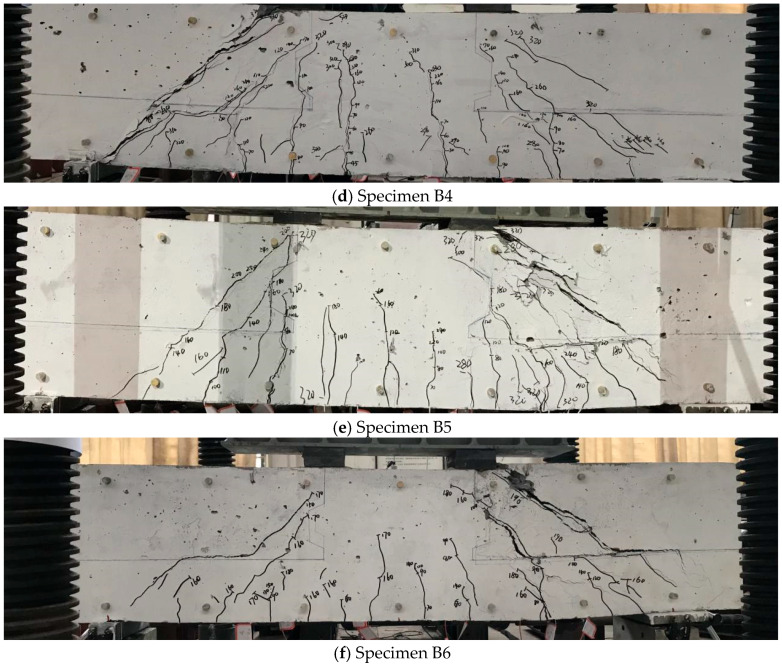

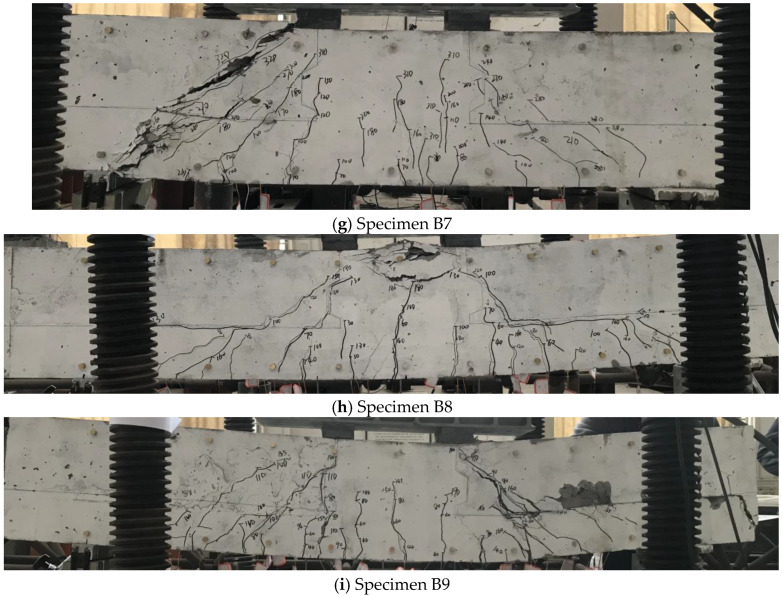
Failure of the specimens and cracks propagation.

Table 5 summarizes the primary test results, including the first-cracking loads, maximum loads, corresponding moments, and dominant failure modes. The final failure patterns and crack propagation characteristics of all specimens are illustrated in Figure 7.

Based on visual inspection and strain data collected during testing, specimens B1 and B8 exhibited flexural-dominated failure modes. Specimens B2 and B3 failed due to bond slip of the longitudinal reinforcement within the pure bending region. All remaining composite specimens experienced shear-dominated failure. In general, the incorporation of steel fibers enhanced both the cracking resistance and ultimate load capacity of the composite beams. The observed improvements in flexural behavior can be attributed to the enhanced tension stiffening effect provided by the steel fibers, which improved bond performance and crack control. For beams subjected to significant shear, steel fibers contributed through their crack-bridging ability, especially across inclined shear cracks, thereby improving overall shear performance.

The benchmark monolithic beam B1 performed as anticipated, displaying stable load-deformation characteristics. No premature cracks or reinforcement rupture were observed during the early loading stages. Flexural cracks first developed in the pure bending region at a load of 65 kN, followed by inclined cracks at 100 kN. The beam ultimately failed in flexure, characterized by yielding of longitudinal reinforcement and concrete crushing at the mid-span. At failure (342 kN), the mid-span deflection was 14.08 mm, and the compressive zone depth reached approximately 65 mm.

In Group 1, specimens B2 and B3 used lap-spliced longitudinal reinforcement at the central joint, with a lap length of 420 mm (10d horizontal, 11d vertical) due to geometric constraints. Both specimens showed initial flexural cracking in the pure bending region, at 70 kN (B2) and 120 kN (B3), followed by diagonal cracking at 160 kN and 130 kN, respectively. Their failure was governed by the bond slip between the lap-spliced bars. Despite this, B2 achieved a peak moment capacity nearly equal to B1, suggesting that lap splice behavior is influenced by the shear span ratio and shear force level.

In Group 2, specimens B4 and B5 differed only in the material of the lower post-cast section (Part B), with B5 incorporating SFRC. Both beams failed in shear, with notable cracking observed at the horizontal joint interface. Concrete in B4 was more severely damaged than in B5, indicating the beneficial role of SFRC. The maximum shear capacity of B5 was approximately 2% higher than that of B4, which falls within the experimental variability; thus, the placement of SFRC in the bottom tensile zone had no significant effect on shear strength under the present test conditions.

In Group 3, specimens B6 and B7 were constructed without stirrups. SFRC was used in the upper compressive zone (Part B). Both specimens failed in shear. In B6, which had normal concrete in compression, damage was concentrated above the horizontal joint, where large joint openings and web cracking were observed due to the absence of stirrup-induced dowel action. In contrast, B7 (fully SFRC) exhibited smaller crack openings and less extensive damage, despite also lacking stirrups. Remarkably, the ultimate load capacity of B7 was comparable to that of B4 and B5, which contained conventional shear reinforcement. These results demonstrate that placing SFRC in the upper compressive region effectively enhances shear resistance in the absence of stirrups. Cracking above the horizontal joint was a key indicator of shear failure initiation.

In Group 4, specimens B8 and B9 had a shear span ratio of 2.4. B8 exhibited typical flexural failure with crushing in the upper concrete of the pure bending zone (Figure 7h). With the increased shear span, longer horizontal cracks developed along the joint interface. The damage zone extended deeper into the compression region compared to B1, reaching approximately 90 mm (versus 65 mm in B1). For B9 (Figure 7i), flexural cracking was less prominent, but significant horizontal joint separation was observed at the interface between SFRC and normal concrete, particularly on the shear-dominated side. SFRC in the lower region helped delay failure, but large slip and crack openings indicated insufficient integrity at the joint. The upper SFRC remained intact, confirming its protective role in the compression zone.

Overall, the test results suggest that horizontal joints in precast composite beams significantly influence shear and flexural performance. Unlike monolithic cast-in-place members, precast specimens exhibited discontinuous critical shear cracks and less uniform crack width distribution. Placement of SFRC in the upper compressive region demonstrated a more pronounced benefit to shear resistance than in the lower tensile zone.

### 3.2. Load-Displacement Curves and Ductility

**Figure 8:**
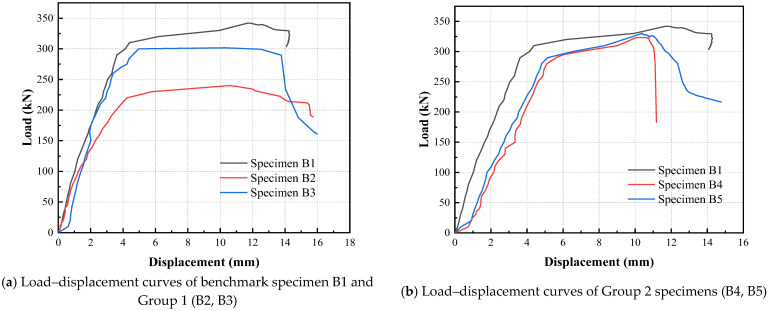

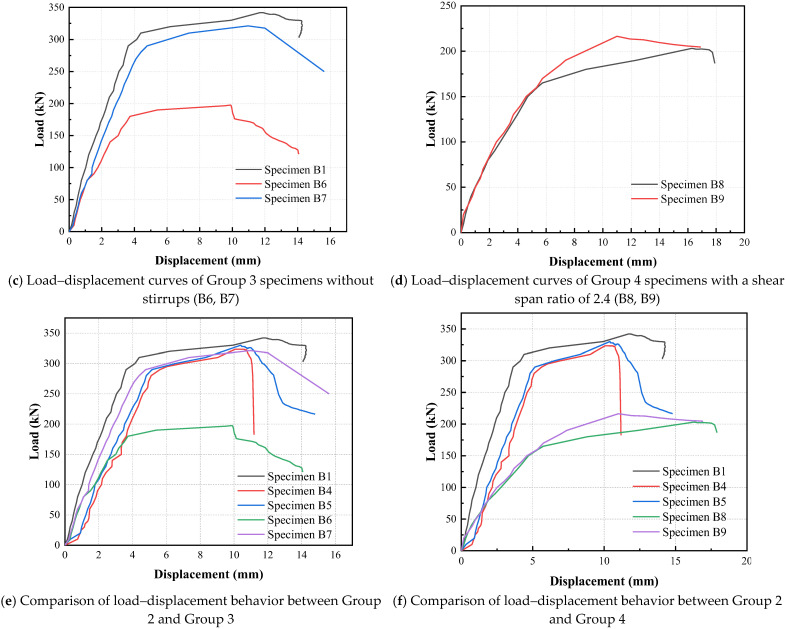
Load–displacement curves of tested composite beams.

Figure 8 presents the load–displacement responses of all tested specimens. The curves for B1, B2, and B3 are shown in Figure 8a. Specimen B1, serving as the cast-in-place benchmark with continuous longitudinal reinforcement, demonstrated excellent stiffness and load-bearing capacity owing to its integral monolithic configuration. The load–displacement curve of B1 exhibited the typical stages of flexural failure: initial cracking marked by a reduction in stiffness, crack propagation with further stiffness degradation, yielding of longitudinal reinforcement resulting in a more gradual load increase, and finally, concrete crushing in the compression zone. In contrast, specimens B2 and B3 experienced bond–slip failure following reinforcement yielding. After yielding, a reduction in bond stress and a significant increase in slip deformation were observed, particularly near the ends of the lap splice, where large crack widths developed. Due to a larger shear span ratio, specimen B2 exhibited reduced stiffness and lower peak load compared to B1 and B3.

Figure 8b illustrates the load–displacement curves of Group 2 specimens (B4 and B5). Both beams were subjected to shear failure. B5, incorporating SFRC in the lower tensile region, displayed slightly higher stiffness and peak load relative to B4, which can be attributed to enhanced tension stiffening provided by the steel fibers. However, the post-peak slope of B4 was noticeably steeper, indicating a more brittle failure process. The milder degradation in B5 suggests that residual tensile bridging by steel fibers mitigated sudden slip at crack interfaces. Despite this, both specimens demonstrated relatively low ductility and deformation capacity when compared to B1. This highlights that precast composite beams, even when enhanced with fibers, tend to exhibit inferior deformation behavior compared to monolithic counterparts when dominated by shear.

Figure 8c displays the response of Group 3 specimens, B6 and B7, both of which were constructed without stirrups. B7, which employed SFRC in both compression and tension zones, outperformed B6 significantly, achieving approximately 63% higher peak load. This result confirms the substantial contribution of steel fibers to shear resistance in the absence of conventional stirrups. Notably, the stiffness of both beams was comparable in the initial stages; however, B7 exhibited improved ductility, evidenced by a less steep post-peak slope relative to B6 and to Group 2 specimens. The test results suggest that SFRC with a volume fraction $V_{f}$ = 1.0 can provide a shear resistance equivalent to HRB400 stirrups with 8 mm diameter spaced at 200 mm.

The load–displacement behavior of Group 4 (B8 and B9) is presented in Figure 8d. B8 failed in flexure while B9 failed in shear. Before yielding, both specimens exhibited similar load–displacement trends. Differences became more pronounced during the post-yielding phase. The concrete in B8’s compression zone failed prematurely, resulting in a lower peak load. However, due to substantial slip along the horizontal interface between Parts A and B, the deformation capacity of B9 was not superior to that of B8 despite its higher load-bearing capacity. These observations suggest that in composite beams with relatively large shear span ratios, adequate transverse reinforcement is essential to control slip and crack propagation at horizontal joints. The importance of stirrup reinforcement in such configurations is further emphasized when compared to Group 3, where beams without stirrups but with smaller shear span ratios exhibited less severe joint degradation. This conclusion is reinforced by Figure 8e and Figure 8f, which provide comparative curves between Groups 2 and 3, and Groups 2 and 4, respectively.

Table 6 lists the yielding displacement, ultimate displacement, and calculated ductility factors of all specimens. Ductility was evaluated using a graphical method (farthest point method). For specimens B2 and B3, the values of yield load *F_y_* and displacement *δ_y_* do not correspond to actual reinforcement yield points, but rather to the onset of bond–slip behavior. Except for B6, which showed limited shear resistance due to the absence of stirrups and incomplete SFRC coverage, most composite beams that failed in shear demonstrated significantly lower ductility than B1. The presence of joints adversely affected structural integrity, thereby reducing shear capacity. Nevertheless, placing SFRC in the upper compression region substantially enhanced the ductility, even for specimens without any transverse reinforcement. Of particular interest is specimen B8, which achieved comparable strength, deformation capacity, and ductility to B1, despite being a precast configuration. This further supports the efficacy of SFRC in enhancing composite member performance under complex loading conditions.

### 3.3. Longitudinal Strain

**Figure 9:**
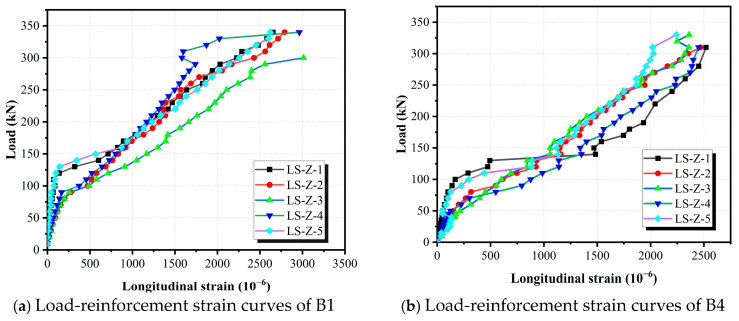

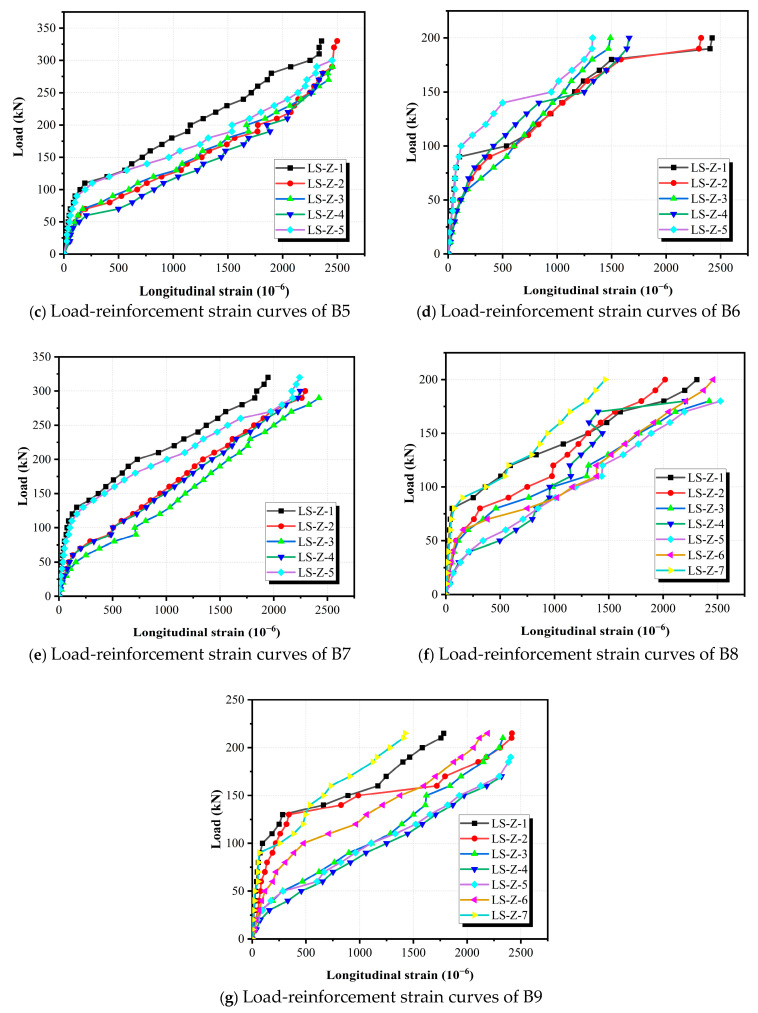
Load–reinforcement strain curves of composite beams.

During the tests, static strain gauges were employed to monitor the longitudinal strain development in the reinforcement, with the aim of assessing the influence of steel fibers on bar strain behavior. The specific locations and distribution of the strain gauges are depicted in Figure 6a. The measured load–strain relationships for selected specimens are presented in Figure 9. Note that several gauges failed after the yielding of the steel bars, and data beyond the measurement range have been excluded from the figure.

Overall, the strain in the longitudinal reinforcement exhibited a gradual increase prior to the initiation of concrete cracking. Once cracking occurred, particularly in the pure bending region, strain values increased rapidly. Typically, initial cracking was observed at the mid-span where the flexural moment was greatest, followed by cracking in the shear span regions. As cracks developed, the corresponding bar strains at those locations increased accordingly.

For beams B1 and B8, both of which exhibited flexural failure modes, yielding of the longitudinal reinforcement occurred during the latter stages of loading. Yielding was primarily driven by flexural demand, with the yielding location typically falling within or adjacent to the pure bending zone. Among the recorded data points, the strain gauge LS-Z-3 positioned at the mid-span captured the highest strain values, consistent with the expected flexural behavior.

In contrast, for the majority of specimens that failed in shear, the longitudinal reinforcement remained largely within the elastic range throughout the loading process. In these cases, the maximum measured strain did not exceed 2500 με, corresponding to the yield strain associated with a bar yield strength of approximately 502 MPa. While some bars approached yielding at advanced stages of loading, most remained below this threshold. The simultaneous action of flexural moment and shear stress contributed to an increase in bar strain, especially near the critical shear crack zones. Notably, the strain levels recorded at LS-Z-1 or LS-Z-5—located near the ends of the beam—were comparable to those at LS-Z-2, LS-Z-3, and LS-Z-4, despite being in regions of lower bending moment. This phenomenon is likely due to the dowel action exerted by the longitudinal reinforcement as it bridged across developing shear cracks.

In the case of B9, the strain gauge was damaged during the late stage of loading at approximately 190 kN. At that time, the recorded strain had nearly reached the yield strain limit of 2500 με. Although no further data could be collected beyond this point, visual observation of the specimen revealed the development of a wide crack (exceeding 1 mm) in the shear–flexural region at the beam bottom, corroborating the approach to bar yielding prior to failure.

The interpretation of reinforcement strain results was further extended by comparing the experimental observations with theoretical expectations. In shear-dominated specimens, the longitudinal reinforcement remained mostly elastic, consistent with the STM prediction that diagonal struts and ties primarily govern shear transfer. In flexure-dominated specimens, the longitudinal bars yielded at mid-span, validating the applicability of the sectional assumption. Moreover, SFRC specimens exhibited lower reinforcement strains compared with NC counterparts, which reflects the tension-stiffening effect of fibers and agrees with previously reported findings. Overall, the observed strain development is in good agreement with theoretical models, supporting the reliability of the adopted analysis approaches.

### 3.4. Strain of Stirrups

The measurement of stirrup strain serves not only to determine whether yielding has occurred, but also to verify the relative positions of critical shear cracks and to assess the influence of steel fibers on stirrup stress responses.

**Figure 10:**
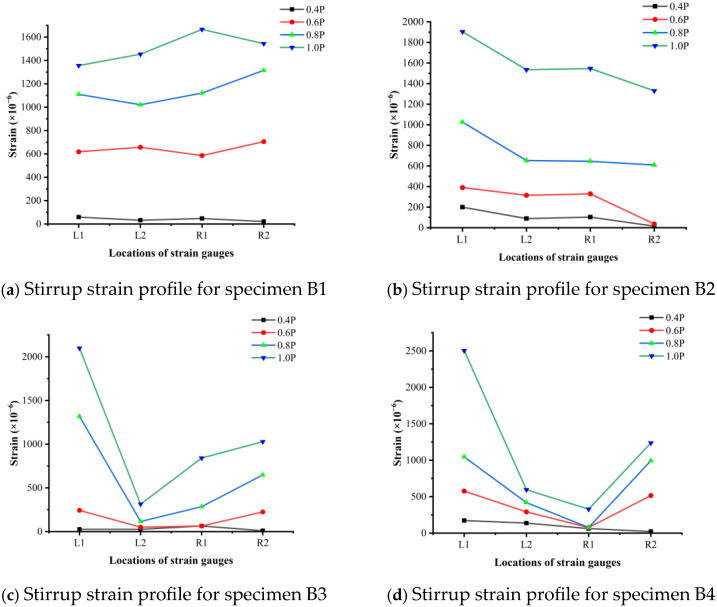

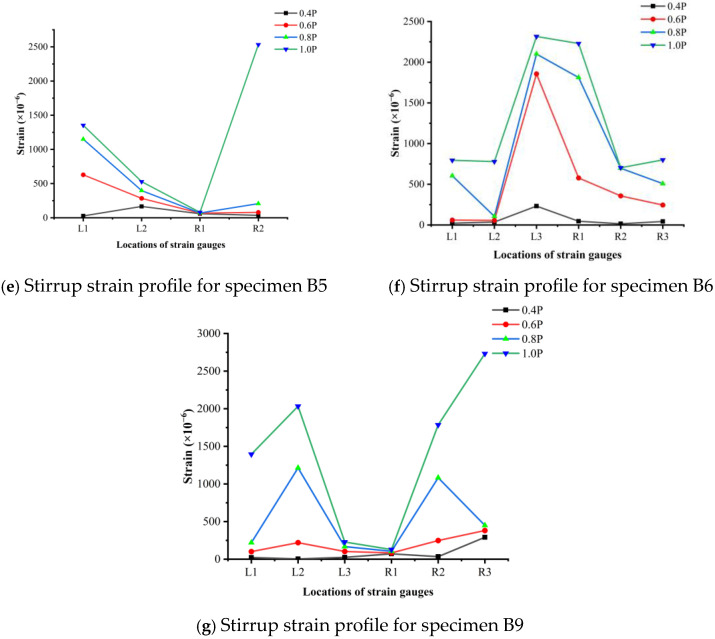
Stirrup strain distributions in tested beams under various load levels.

In this study, four strain gauge locations—L1, L2, R1, and R2—were installed on the surface of the stirrups in each specimen (with additional points L3 and R3 for B8 and B9), as shown in Figure 6a. Measurement points L1 and R2 were located in the shear–flexural zones, while L2 and R1 were placed in the pure bending regions (except in specimen B2, where all zones were modified due to lap splicing). The stirrup strains corresponding to 40%, 60%, 80%, and 100% of each specimen’s maximum load (denoted as 0.4P, 0.6P, 0.8P, and 1.0P, respectively) are presented in Figure 10.

In the benchmark cast-in-place beam B1 and the composite beam B8—both of which failed due to compressive crushing of the concrete—the stirrup strains in the pure bending regions were relatively moderate, as illustrated in Figure 10a,f. None of the strain measurements at these locations exceeded the yielding threshold under the maximum applied load. This is consistent with the flexural failure mode identified earlier for B1, where no inclined shear cracks were observed intersecting the stirrups. Consequently, the stirrups in the pure bending region may have functioned primarily to confine the concrete and enhance its compressive behavior.

For composite specimens B4 and B5, both of which experienced shear-compression failure, the stirrups located in the region of the critical shear crack yielded, with measured strains surpassing 2500 με, as shown in Figure 10d,e. In B4, the critical inclined shear crack developed progressively with load, while in B5, a sudden spike in stirrup strain indicated abrupt crack formation and a more brittle failure mechanism. These contrasting behaviors highlight the variable nature of shear cracking in composite systems.

In specimen B9, which failed under a combination of flexural effects and horizontal joint slipping, the stirrup strain at measurement points L3 and R1—located in the pure bending zone—remained low (below 200 με). However, the strains at L1, L2, R2, and R3 were significantly larger. This was attributed to the substantial opening of the horizontal joint between the normal concrete and the SFRC regions, which induced elevated stress concentrations in the adjacent stirrups.

The observed stirrup strain distributions underscore the importance of crack location and joint integrity in determining internal force redistribution. Given the complex nature of these stress fields, additional strain gauges distributed across a wider range of locations could provide more comprehensive insights into stirrup performance under combined flexural–shear actions in composite members.

### 3.5. Local Deformation of the Concrete

**Figure 11:**
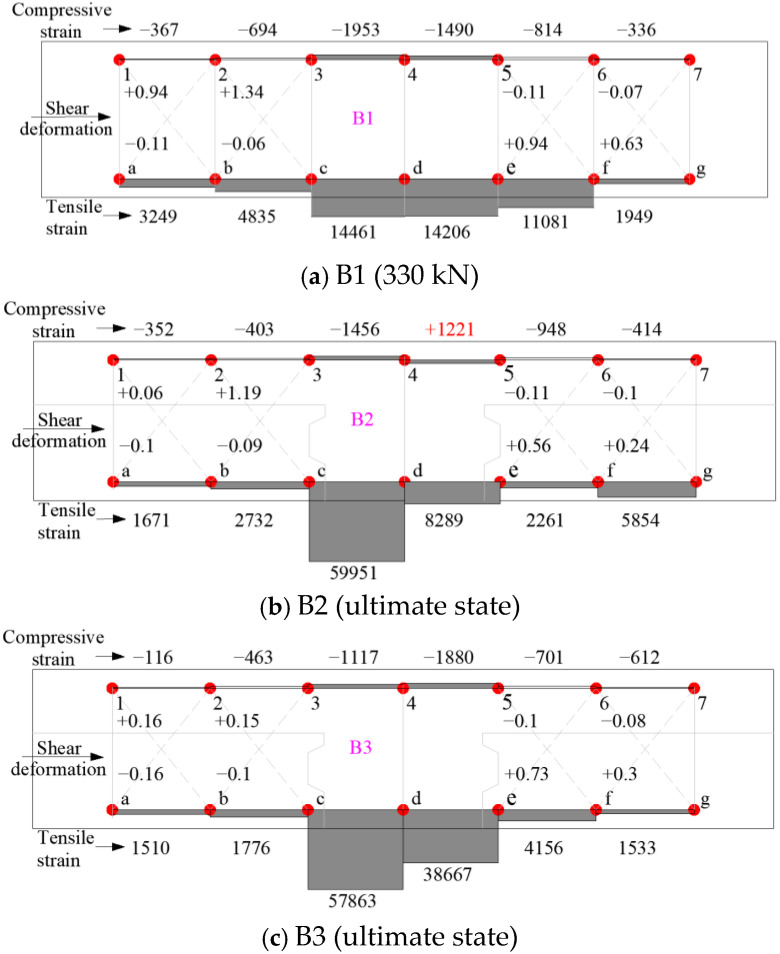

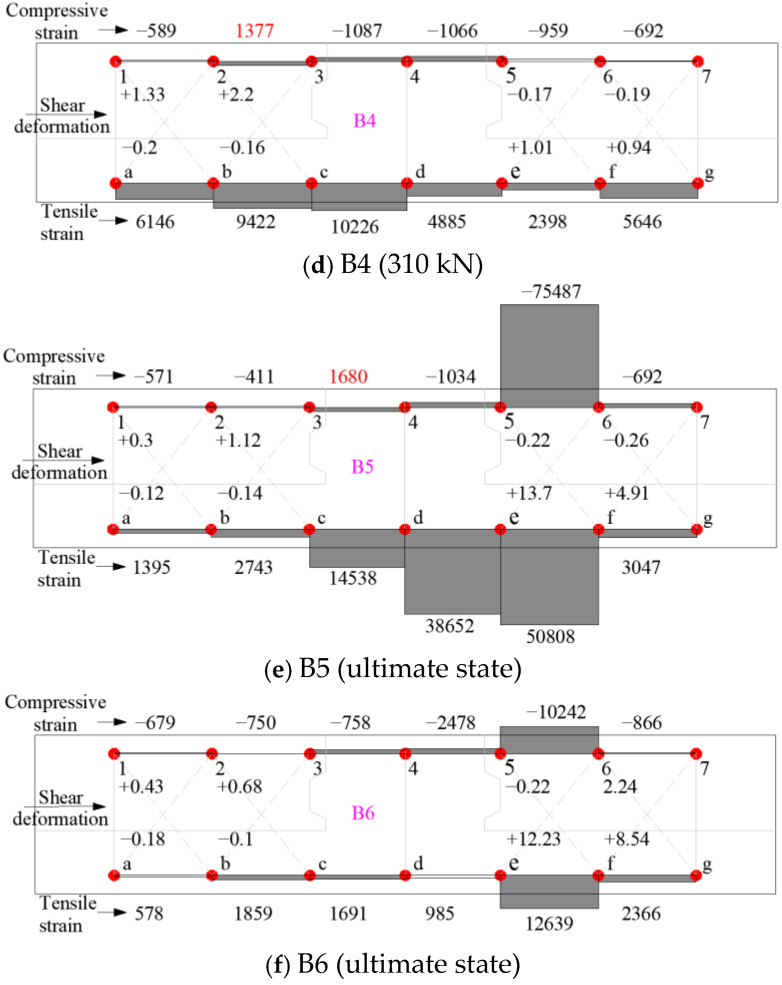

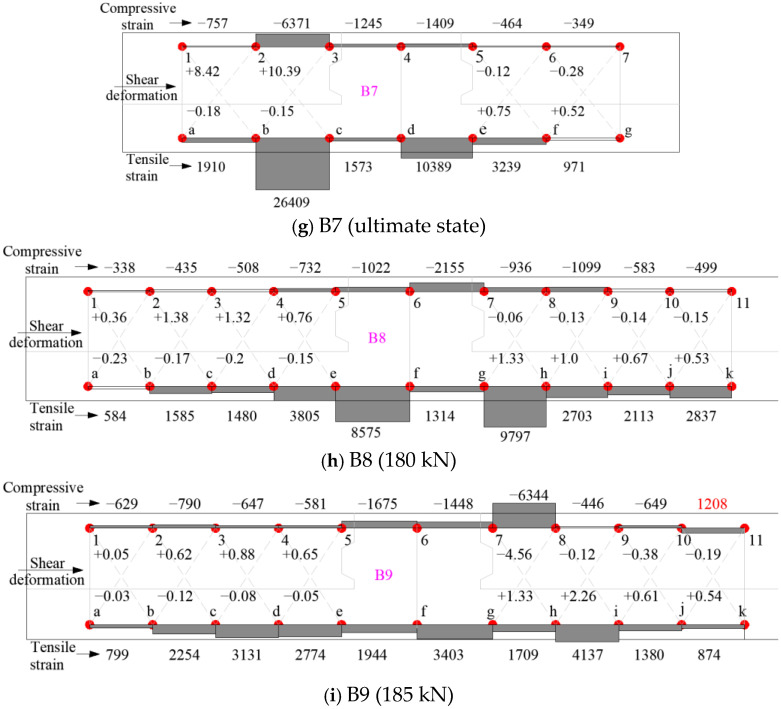
Compressive/tensile strains and shear deformation patterns of the concrete.

The analysis of local concrete deformations provides intuitive insights into the actual damage evolution and failure mechanisms of the tested specimens. The measurement points for concrete strain were arranged as shown in Figure 6. The average compressive strain (με), average tensile strain (με), and shear deformation (mm) at the ultimate loading stage are illustrated in Figure 11. Each beam was subdivided into six or ten planar elements, such as element 12ba or 23cb. Typically, the upper portion of the beam was subjected to compression (with negative strain values), while the lower portion was under tension (with positive strain values). However, in specimens with small shear span ratios, the upper concrete might experience a combination of compressive and shear stresses, resulting in strain values that are not necessarily negative, as observed in Figure 11d,e.

In specimens B2 and B3, which failed due to bond–slip behavior at the lap splice regions, large local deformations occurred at the spliced zone. This was caused by relative displacement between the lap-spliced longitudinal bars. In contrast, the largest tensile or compressive strains in shear-failed specimens B4, B5, B6, and B7 were generally concentrated along the critical shear crack path, where significant shear deformation was observed.

For regions subjected to combined shear and bending, such as elements 12ba, 23cb, 56fe, and 67gf, the displacements along directions 1b, 2c, 5f, and 6g—approximately orthogonal to the compressive struts—were positive. These directions correspond to the tensile ties in a strut-and-tie model. Notably, the deformation magnitudes of these tensile tie zones in the composite beams were significantly greater than those observed in specimen B1. This suggests that the presence of horizontal joints, which typically have poor bond performance, weakens the strut-tie action and adversely affects shear resistance.

However, the incorporation of steel fibers into the compressive zones was shown to enhance the strut behavior. Owing to the superior compressive ductility, tensile strength, and post-cracking resistance of SFRC, the performance of both struts and ties can be significantly improved when SFRC is used in the compression zone.

For specimens B8 and B9 with larger shear span ratios, the horizontal joint likewise influenced shear behavior. The joint acted as a weak plane separating the upper and lower concrete sections. In these specimens, only the upper concrete exhibited crushing and substantial compressive deformation, highlighting the decoupling effect of the horizontal interface on stress transfer between structural components.

## 4. Bearing Capacity Model for the Composite Beams

The analysis and design of shear in concrete structures remain subjects of ongoing debate due to the inherent complexity of shear behavior. ACI 318-14 code [36] recommends the use of the strut-and-tie model (STM) for analyzing and designing any structural concrete member that contains a disturbed region (D-region), such as deep beams, corbels, beam–column joints, diaphragms, and shear walls. This model originates from the truss analogy, which provides an effective framework for simulating force transmission paths in discontinuous or non-uniform regions of concrete members. For B-regions (beam regions, where Bernoulli’s assumption is valid), conventional sectional shear design procedures are typically adopted.

Although both methods have been widely applied in structural design, there remains a lack of literature comparing their computational predictions, particularly when applied to composite concrete members. In composite beams, the existence of horizontal or vertical joints may reduce both the flexural and shear capacity relative to monolithic counterparts. This reduction becomes more uncertain when the longitudinal reinforcement ratio and the shear span ratio fall within intermediate ranges (e.g., 1.5 to 2.5), where failure mechanisms may transition between flexure- and shear-dominated modes.

To address these complexities, three modeling approaches are employed in this study to estimate the bearing capacities of the tested composite beams:

(1) The fiber beam–column element model, based on the plane section assumption, is used to estimate the flexural capacity;

(2) The sectional shear design method, following the ACI 318-14 provisions, is used to compute the shear strength;

(3) The strut-and-tie model is applied to assess the global bearing capacity.

### 4.1. Flexural Bearing Capacity Using Fiber Beam–Column Element Model

**Figure 12:**
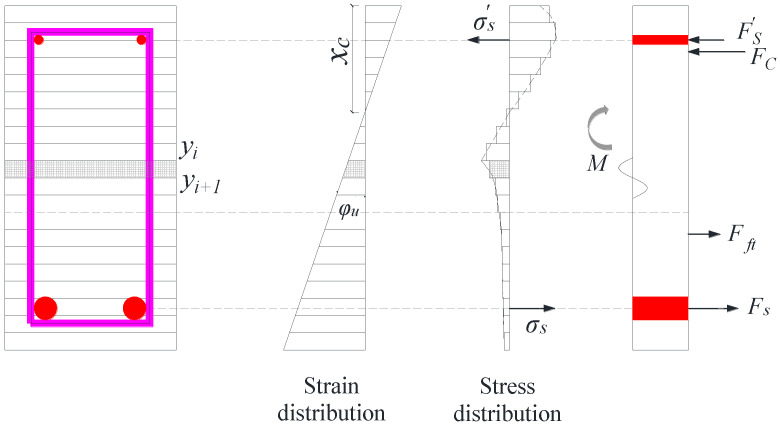
Schematic description of stress and strain distributions in cross-section.

To estimate the flexural strength of the composite beams, a fiber-based beam–column element model was employed under the assumption that the joint interface between the precast and post-cast concrete segments can effectively transfer shear. The cross-section was discretized into a series of longitudinal fibers, each governed by uniaxial constitutive laws. The distribution of strain across the section was calculated using the plane section assumption, as schematically illustrated in Figure 12.

Each fiber—either steel or concrete—follows an independent stress–strain relationship. The flexural strength of the cross-section is computed by integrating the axial stresses and moments in each fiber as follows:

(2) $$\;V_{M}=\left(\sum_{i=1}^{m}f_{si}A_{si}y_{si}+\int_{0}^{h}bf_{c}(y)ydy\right)/a=\;\left(\sum_{i=1}^{m}f_{si}A_{si}y_{si}+\sum_{j=1}^{n}f_{cj}A_{ci}y_{cj}\right)/a$$

It is subject to the axial force equilibrium condition as follows:

(3) $$\;\sum_{i=1}^{m}f_{si}A_{si}+\sum_{j=1}^{n}f_{cj}A_{ci}=0\;\;\;\;\;\;\;\;\;\;\;\;\;\;\;\;\;$$

where $f_{si}$, $A_{si}$ and $y_{si}$ are the stress, the cross-section area of fiber i of longitudinal reinforcement, and the distance from the center of each longitudinal reinforcement fiber to the center of the cross section, respectively; m and n are the numbers of fibers of the longitudinal bar and concrete, respectively; b is the beam width; and $f_{c}(y)$ (or $f_{cj}$) and y (or $y_{cj}$) are the stress of the concrete fibers and the distance from each concrete fiber to the center of the section, respectively.

Regarding the compressive behavior of SFRC, the compressive stress–strain relationship of SFRC was modeled using the formulation proposed by Gao [37], which considers the contribution of steel fibers to the descending branch as follows:

(4) $$\;y=\left\{\begin{array}{cc} ax+\left(3-2a\right)x^{2}+\left(a-2\right)x^{3}, & 0\leq x\leq 1\\ x/\left[R{\left(x-1\right)}^{2}+x\right], & x>1 \end{array}\right.$$

(5) $$a=E_{fc}\left(1.3+0.014f_{fc}+0.96V_{f}l_{f}/d_{f}\right)/\left(f_{fc}\times 10^{3}\right)$$

(6) $$R=\left(1.4+0.012f_{fc}^{1.45}\right)\left[1-0.8{\left(V_{f}l_{f}/d_{f}\right)}^{0.295}\right]$$

(7) $$x=\varepsilon /\varepsilon_{f0}$$

(8) $$y=\sigma /f_{fc}$$

$\;E_{cf}$ is taken as

(9) $$\;E_{cf}=\frac{10^{5}}{2.2+\left(34.7/f_{cu,k}\right)}\left(1-0.0006\lambda_{f}\right)$$

where $f_{fc}$ is the compressive strength of SFRC; $f_{cu,k}$ is the standard cube compressive strength of SFRC; $\varepsilon_{f0}$ is the peak compressive strain of SFRC related to the compressive strength $f_{fc}$; and $\varepsilon_{f0}$ can be expressed as

(10) $$\;\varepsilon_{f0}=\left(1.3+0.014f_{fc}+0.96V_{f}l_{f}/d_{f}\right)\times 10^{-3}$$

(11) $$\lambda_{f}=V_{f}l_{f}/d_{f}$$

where $V_{f}$, $l_{f}$, and $d_{f}$ are the volume ratio, length and diameter of steel fiber, respectively.

The composite beam section is divided into an SFRC layer and an ordinary concrete layer. Steel fibers enhance the tensile strength and soften the descending branch of the stress–strain curve, thereby improving stiffness in the tension zone. To better reflect their contribution, the tensile strength of SFRC in the lower part of the section is considered. The uniaxial tensile stress–strain behavior is defined by ascending and descending segments, as proposed in [38], as follows:

(12) $$y=\left\{\begin{array}{cc} \frac{x}{0.33x^{3.03}+0.67}, & 0\leq x\leq 1\\ \frac{x}{\alpha_{2}{\left(x-1\right)}^{\beta_{2}}+x}, & x>1 \end{array}\right.$$

(13) $$x=\varepsilon /\varepsilon_{p}$$

(14) $$y=\sigma /\sigma_{p}$$

where $\varepsilon_{p}$ and $\sigma_{p}$ are the peak axial tensile strain and peak axial compressive stress of SFRC, respectively; σ and ε are the axial tensile stress and axial tensile strain of SFRC, respectively; and $\alpha_{2}$ and $\beta_{2}$ are the coefficients related to matrix and steel fiber properties, where $\beta_{2}$ can be taken as 1.7. This tensile model captures the post-cracking behavior and the bridging effect of the steel fibers, which significantly enhance the stiffness and toughness in the tension zone.

(15) $$\;\alpha_{2}=0.22\times f_{fc}\times {\left(1+\lambda_{f}\right)}^{-1.74}$$

### 4.2. Shear Strength Based on Section Shear Design Method

According to ACI 318-14 [36], the vertical shear strength of reinforced concrete beams can be estimated using the following expressions:

(16) $$\;V_{c1}=0.167\sqrt{f_{c}^{\prime }}b_{w}d$$

(17) $$V_{c2}=\left(0.16\sqrt{f_{c}^{\prime }}+17\rho \frac{V_{u}d}{M_{u}}\right)bd\;\;\;$$

where $V_{c2}$ ≤ $0.29\sqrt{f_{c}^{\prime }}b_{w}d$; $V_{u}/M_{u}$ ≤ 1.0; $f_{c}^{\prime }$ is the compressive strength of the concrete (MPa); b is the web width (mm); d is the distance from the extreme compression fiber to the centroid of the flexural reinforcement in tension (mm); ρ is the flexural reinforcement ratio (=$A_{s}/b_{w}d$); $V_{u}$ is the factored shear force at the section (N); and $M_{u}$ is the factored moment at the section (N-mm).

For SFRC beams, ACI 544 provides the following expression for the vertical shear strength:

(18) $$\;V_{cf}=0.667f_{t}^{\prime }\left(\frac{d}{a}\right)bd$$

where $f_{t}^{\prime }$ is the tensile strength of concrete; and d/a is the ratio of effective depth to shear span. $f_{t}^{\prime }$ can be approximated based on the empirical model proposed by Thomas (2007) [39] as follows:

(19) $$\;f_{t}^{\prime }=0.63\sqrt{f_{c}^{\prime }}+0.288\lambda_{f}\sqrt{f_{c}^{\prime }}+0.052\lambda_{f}$$

For RC beams with transverse stirrup, the shear strength provided by the stirrup $V_{s}$ can be expressed as follows:

(20) $$\;V_{s}=\frac{A_{v}f_{y}d_{s}}{s}\;\;\;\;\;\;\;\;$$

where $A_{v}$ is the stirrup area; s is the stirrup spacing; $f_{y}$ is the yield strength of stirrup; and $d_{s}$ is the stirrup diameter.

For composite beams, the presence of horizontal construction joints significantly affects the overall shear resistance. In cases where the horizontal joints are unreinforced and undergo shear slippage or failure, the nominal shear strength can be approximated as follows:

(21) $$\;V_{nh1}=0.55b_{v}d_{v}\;$$

When horizontal joints are damaged, the shear strength of composite beams with transverse stirrups can be expressed as follows:

(22) $$\;V_{nh2}=\left(1.8+0.6\rho_{v}f_{y}\right)\lambda b_{v}d_{v}\;$$

where $\rho_{v}$ is the ratio of the cross-sectional area of transverse stirrups to the interface area ($\rho_{v}$ = $A_{v}/b_{v}s$); and $b_{v}$ and $d_{v}$ are the width and length of the cross-section at the interface (mm), respectively. The value of λ is related to the concrete type.

The shear strength of the beam transformed by shear failure of the horizontal joint of the composite beam is expressed as follows:

(23) $$\;V_{h}=0.9V_{nh}d/a$$

where $V_{nh}$ is the horizontal shear strength of horizontal joint (=$V_{nh1}$ or $V_{nh2}$); and a is the shear span.

### 4.3. Shear Strength Based on Strut-and-Tie Model

**Figure 13:**
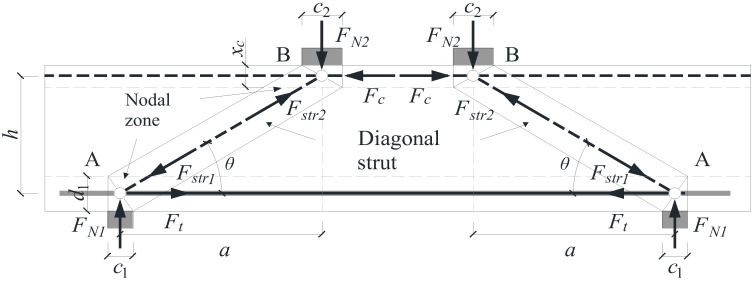
Standard strut-and-tie model.

The ACI 318-14 [36] code recommends the use of the strut-and-tie model (STM) for the design of disturbed regions (D-regions) in reinforced concrete members. However, limited research has focused on applying STM to analyze the shear behavior of composite beams, especially in the presence of construction joints. In this study, a standard strut-and-tie model is developed for the composite beam, as illustrated in Figure 13.

In the proposed model, the effects of vertical and horizontal construction joints are neglected for simplification. The geometry of the STM includes two critical nodes: Node A at the support region and Node B at the loading region. Each node contains an internal tie, compressive strut, and nodal zone. The total shear resistance is derived from the contributions of the associated struts and ties.

For Node A, the corresponding ultimate shear resistances are computed as follows:

(24) $$\;V_{N1}=F_{N1}=\beta_{n}f_{c}^{\prime }c_{1}b\;$$

(25) $$V_{tie}=F_{t}\tan\theta =A_{s}f_{y}\tan\theta \;$$

For Node B, the corresponding ultimate shear resistances are as follows:

(26) $$\;V_{N2}=F_{N2}=\beta_{n}f_{c}^{\prime }c_{2}b\;$$

(27) $$V_{Fc}=F_{c}\tan\theta =bx_{c}f_{c}^{\prime }\tan\theta \;$$

(28) $$V_{str2}=F_{str2}\sin\theta =bh\beta_{s}f_{c}^{\prime }\sqrt{x_{c}^{2}+c_{2}^{2}}/\sqrt{a^{2}+h^{2}}\;$$

where $V_{N1}$, $V_{tie}$, and $\;V_{str1}$ are the shear strengths calculated from the node strength of node 1 (or node *A*) and nearby vertical support surface strength $F_{N1}$, horizontal tie $F_{t}$, and diagonal strut strength $F_{str1}$, respectively; and $V_{N2}$, $V_{Fc}$, and $\;V_{str2}$ are the shear strengths calculated from the node strength of node 2 (or node *B*) and nearby vertical loading bearing surface strength $F_{N2}$, horizontal strut $F_{c}$, and diagonal strut strength $F_{str1}$, respectively. $\beta_{n}$ and $\beta_{s}$ are the strength reduction factors, which shall be in accordance with ACI 318-14. The effective strength of 0.85 $f_{c}^{\prime }$ of concrete under long-term compression is not considered. x is the depth of the concrete compressive zone; and $x_{c}$ is the depth of the equivalent rectangular stress block in the compressive zone. θ is the angle between the axes of the strut *AB* and the horizontal tie entering the single node *A*, which can be calculated as follows:

(29) $$\;\theta =\tan^{-1}a/h\;$$

The final shear capacity $V_{st}$ predicted by the STM is taken as the minimum of all individual shear contributions calculated in Equations (23) to (28) as follows:

(30) $$\;V_{st}=min\left\{V_{N1},V_{tie},V_{str1},V_{N2},V_{Fc},V_{str2}\right\}\;$$

### 4.4. Predicted Moment and Shear Resistance in Comparison with Test Results

The predicted flexural and shear strengths of all specimens based on theoretical models are summarized in Table 7, alongside experimental results for direct comparison. The comparisons of experimental shear/flexural capacities and model predictions are summarized in Table 8.

From the results, it is evident that the existence of horizontal joints in composite beams influenced both flexural and shear behavior. When the influence of joints was not explicitly considered, the theoretically predicted flexural capacities, accounting for 1% steel fiber volume, differed by up to 5%. This indicates that steel fibers primarily enhance the post-peak compressive behavior of concrete by smoothing the descending portion of the stress–strain curve, though their impact on peak compressive strength is limited.

However, the tensile performance of steel fiber-reinforced concrete (SFRC) was significantly better than that of plain concrete. In the flexural tension zone, SFRC contributed approximately 8.5% of the total tensile force compared to the reinforcement, effectively enhancing the stiffness of the tension region. As such, placing SFRC in both the compression and tension zones of the section led to a moderate increase in the flexural capacity. For example, specimens B2 and B5, with SFRC in critical zones, exhibited higher predicted flexural strength than B1 and B4.

It was observed that the actual load-bearing capacities of all specimens were generally lower than their predicted flexural capacities. This discrepancy may be attributed to experimental uncertainties, small shear span ratios (which place the critical region within the D-region and thus violate the plane section assumption), actual material properties, and the effect of construction joints. Notably, the shear strengths predicted by ACI 318-14 were conservative for most specimens, underestimating the observed values. Similarly, shear strengths derived from horizontal joint failure models were also conservative, especially for specimens with a shear span ratio of 1.5 and no stirrups.

The results confirmed that shear span ratio strongly influences the flexural and shear performance of composite beams, and that horizontal joints reduce shear capacity more severely in members with longer spans. However, the impact of vertical prefabricated joints appeared limited under the observed failure modes, which were dominated by diagonal cracking and concrete crushing.

To further validate the shear resistance model, the results calculated using the strut-and-tie model (STM) are listed in Table 9.

The predicted shear strengths $V_{st}$ derived from the minimum of components $V_{N1}$, $V_{tie}$, $V_{str1}$, $V_{N2}$, $V_{Fc}$, and $V_{str2}$ generally aligned well with experimental data. The STM predictions were typically governed by the diagonal strut contribution $V_{str1}$, and the results closely matched the measured shear strengths. However, the STM does not distinguish between compression and tension placement of steel fibers, and, thus, could not account for the experimentally observed differences between B6 and B7, where fiber distribution clearly affected behavior.

For specimens with shorter shear spans, the predicted values of both V and $V_{st}$ yielded conservative results with adequate safety margins. In contrast, for specimens with longer shear spans, the predictions were closer to or slightly less than experimental values, which implies a reduction in model safety. Notably, composite beams with longer shear spans exhibited larger deformations at the horizontal joint, confirming that shear transfer across the joint becomes more critical as the shear span increases.

Therefore, although STM provided satisfactory predictions overall, refinement is necessary to include the effects of steel fiber distribution and joint behavior—particularly for composite beams with large shear spans or limited transverse reinforcement.

Although the present program included only nine specimens, some indicative parametric trends can be identified from the combined experimental and analytical results. Beams with larger shear span ratios (B8–B9) exhibited more pronounced joint slip and reduced ductility compared with those with lower a/d ratios, while specimens incorporating SFRC in the compression zone consistently achieved higher shear resistance and deformation capacity than those with fibers only in the tensile zone. These observations highlight the importance of the shear span ratio and fiber distribution as governing parameters for the global performance of SFRC composite beams with joints. A more systematic parametric study, particularly on fiber volume fraction, will be pursued in future research, building on the validated modeling frameworks presented here.

In summary, the analytical validation confirmed that the ACI 318 sectional method provided conservative shear strength estimates, especially for short-span beams without stirrups; the fiber beam–column model reproduced the flexural behavior within about ±10% of the test data; and the strut-and-tie model captured the discontinuity effects of horizontal joints with predictions close to the experimental values. These comparisons demonstrate that the analytical approaches not only support the experimental findings but also directly serve the main goals of this study by identifying reliable design and analysis methods for SFRC composite beams with joints.

## 5. Conclusions

In this study, nine composite beam specimens—including both normal concrete and steel fiber-reinforced concrete (SFRC) sections—were experimentally tested and analytically modeled to investigate their flexural and shear–flexural behavior. Based on a comprehensive analysis of the structural responses, including strain, deformation, and failure patterns, the following conclusions can be drawn:

(1) The presence of joints in composite beams, particularly horizontal joints, has a notable impact on both flexural and shear strength. The overall integrity and continuity of composite members were found to be inferior compared to monolithic cast-in-place counterparts. This reduction in performance is likely due to construction-induced weaknesses, such as reduced compactness and lower strength of post-cast concrete in confined spaces. In composite specimens exhibiting shear failure, critical diagonal shear cracks were typically observed, but often interrupted at horizontal joints, indicating limited continuity. Vertical joints were also identified as potential weak zones, frequently exhibiting cracks under advanced loading conditions.

(2) The incorporation of SFRC into composite beams significantly enhanced both flexural and shear capacities. Flexural improvement was due to enhanced compressive behavior and fiber bridging in tension, while placing SFRC above the horizontal joint proved more effective for shear. For example, the fully SFRC beam (B7) showed ~63% higher capacity than the specimen with fibers only in the lower section (B6), and 1% fiber volume provided shear strength roughly equivalent to HRB400 stirrups (D8@200 mm). SFRC specimens also exhibited improved ductility. In terms of serviceability (SLS), most beams satisfied the L/250 deflection limit until close to ultimate failure, confirming adequate stiffness and crack control within permissible limits.

(3) The longitudinal strain measurements demonstrated that, under identical loading conditions, specimens containing steel fibers exhibited lower strain values than those without fibers. This suggests that the presence of steel fibers contributes to a stress-stiffening effect, enhancing the structural stiffness and delaying the onset of yielding in longitudinal reinforcement.

(4) In specimens with relatively large shear span ratios and shear-dominated failure modes, horizontal joint slip was frequently observed. This shear slippage along the horizontal interface significantly compromised the structural integrity of the member, leading to reduced shear resistance and abrupt failure in some cases. These observations reflect the ultimate limit state (ULS) behavior, where horizontal interfaces may govern the load-bearing capacity.

(5) When the ACI 318-14 section shear design method was applied, the calculated shear strengths of most specimens were found to be significantly lower than the experimental values, indicating a conservative design approach. This conservatism was more pronounced in specimens with a shear span ratio of 1.5 and without transverse stirrups. Additionally, the shear strength estimated by considering horizontal joint failure was also conservative. In contrast, the strut-and-tie model provided predictions more consistent with the observed test results. The fiber beam–column model also reproduced flexural behavior within ±10% of the test data. Therefore, for composite beams with complex shear transfer paths and discontinuities, the strut-and-tie model may offer a more reliable design approach than conventional sectional methods.

## Figures and Tables

**Table 1:** Details of tested specimens, including material configuration, stirrup arrangement, and geometry.

Group	No.	Part A	Part B	Stirrup	Length (mm)	Shear Span Ratio
Benchmark	B1	NC	NC	D8@200	1400	1.5
Grout 1	B2	NC	SFRC	D8@200	1400	2.1
	B3	SFRC	SFRC	D8@200	1400	1.5
Group 2	B4	NC	NC	D8@200	1400	1.5
	B5	NC	SFRC	D8@200	1400	1.5
Group 3	B6	NC	SFRC	–	1400	1.5
	B7	SFRC	SFRC	–	1400	1.5
Group 4	B8	NC	NC	D8@200	1860	2.4
	B9	NC	SFRC	D8@200	1860	2.4

**Table 2:** Measured 28-day compressive strength of normal-strength concrete and SFRC from Part A and Part B.

NO.	Age (Days)	Cube Size (mm)	Compressive Strength of Three Concrete Cubes (MPa)	Average Compressive Strength (MPa)
An	28	150 × 150 × 150	29.1, 29.3, 30.2	29.5
As	28	150 × 150 × 150	30.2, 29.7, 30.3	30.0
Bn	28	150 × 150 × 150	29.5, 30.5, 29.6	29.8
Bs	28	150 × 150 × 150	29.8, 30.1, 30.7	30.2

**Table 3:** Amount of each material in each cubic meter of the concrete.

Concrete	Cement (kg)	Water (kg)	Sand (kg)	Coarse (kg)	Fly Ash (kg)	Admixture (kg)	Volume-Weight (kg/m^3^)
C30	388	160	733	1100	69	5.03	2450
SFRC with $V_{f}$ of 1.0%	388	160	715	1072	69	5.03	–

**Table 4:** Mechanical properties of HRB400 steel reinforcement.

Diameter (mm)	Yield Strength (MPa)		Tensile Strength (MPa)	
	Test	Average Value	Test	Average Value
8	410, 412 and 405	409	589, 610, and 608	600
20	503, 511, and 491	502	608, 612, and 592	604

**Table 5:** Summary of mechanical performance and failure characteristics of the tested specimens.

No.	Material of Shear–Flexural Sections		First-Cracking Load (kN)	Maximum Load (kN)	Maximum Moment (kN·m)	Failure Mode
	Upper Part (mm)	Lower Part (mm)				
B1	NC (300 mm)		65	342.0	68.4	Bending
B2	SFRC (120)	NC (180)	70	240.1	66.03	Bond slip
B3	SFRC (120)	SFRC (180)	120	301.7	60.34	Bond slip
B4	NC (180)	NC (120)	45	323.3	64.66	Shear
B5	NC (180)	SFRC (120)	50	330.1	66.02	Shear
B6	NC (180)	SFRC (120)	61	197.6	39.52	Shear
B7	SFRC (180)	SFRC (120)	70	321.2	64.24	Shear
B8	NC (180)	NC (120)	32	203.1	55.85	Bending
B9	NC (180)	SFRC (120)	40	216.4	59.51	Shear

**Table 6:** Displacement and ductility.

No.	Peak Load (kN)	Displacement at Peak Load	Yielded Load *F_y_*	Yielded Displacement *δ_y_*	Ultimate Load *F_u_*	Ultimate Displacement *δ_u_*	Deformation Ductility *μ*
B1	342.0	11.70	310	4.39	302.7	14.08	3.2
B2	240.1	10.56	220	4.24	204.0	15.44	3.6
B3	301.7	10.30	260	3.38	263.5	13.90	4.1
B4	323.3	10.06	280	5.10	274.8	11.10	2.2
B5	330.1	10.55	285	4.90	278.8	12.35	2.5
B6	197.6	9.87	180	3.73	167.9	11.36	3.0
B7	321.2	10.96	290	4.78	273.0	14.60	3.1
B8	203.1	16.28	165	5.74	186.6	17.90	3.1
B9	216.4	10.99	150	4.59	204.6	16.91	3.7

**Table 7:** Moment and shear capacities of the specimens.

No.	$V_{test}$	① $M_{u}$(kN·m)		x(mm)	Cal. $V_{M}$		ACI $V_{c}$					③ $V_{c}$	$V_{s}$	$V_{nh}$ Er%	$V_{h}$ Er%	④ V Er%	$\frac{V}{V_{test}}$	⑤⑥ $V_{st}$ Er%
							NC		SFRC									
		$M_{u1}$	$M_{u2}$		$V_{M1}$ Er%	$V_{M2}$ Er%	② $V_{c11}$	$V_{c1}$	$V_{c21}$	$V_{c22}$	$V_{cf}$							
B1	171	75	77	96	188	193	37	42	–	–	–	42	54	–	–	96	0.56	145
B2	120	77	76	94	140	138	16	18	20	22	44	64	54	215	94	118	1.00	113
B3	151	80	80	90	199	199	–	–	37	41	107	107	54	157	94	161	1.07	146
B4	162	75	77	96	188	193	37	42	–	–	–	42	54	157	94	96	0.59	145
B5	165	77	78	86	193	195	25	27	12	13	35	62	54	157	94	116	0.70	147
B6	99	77	78	86	193	195	25	27	12	13	35	62	–	157	94	62	0.63	118
B7	161	80	80	90	199	199	–	–	37	41	107	107	–	33	20	107	0.66	118
B8	102	75	77	96	119	122	37	42	–	–	–	42	54	246	94	96	0.94	100
B9	108	77	78	86	122	124	25	27	12	13	31	58	54	246	94	112	1.04	101

Note: ① $M_{u1}$ was calculated by Equations (2)–(9) for common concrete whereas $\lambda_{f}$ was taken as 0; $M_{u2}$ was calculated using the constitutive relation of the classic Kent and Park model for common concrete [40,41] without considering the tensile strength; ② $V_{c11}$ and $V_{c12}$ were the shear strengths of plain concrete layer in the composite section without and with consideration of bent reinforcement, respectively; and $V_{c21}$ and $V_{c22}$ were the shear strengths of the SFRC layer in the composite section without and with consideration of bent reinforcement, respectively, calculated by using the plain concrete shear calculation Equations (15) and (16). ③ $V_{c}$ = $V_{c12}$ + $V_{cf}$, where for plain concrete $V_{cf}$ = 0, and for the whole cross-section SFRC $V_{c12}$ = 0; ④  V = $V_{c}$ + $V_{s}$, where for beam without stirrup, $V_{s}$ = 0; ⑤ For $V_{st}$ of B6 and B7 without stirrup, $\beta_{s}$ = 0.6, according to ACI 318-14; and ⑥ For $V_{st}$ of B2, B8 and B9, the inclination angle θ of the diagonal strut may be less than 25 degrees. However, considering the consistency of the strut-and-tie model, the same model was still used.

**Table 8:** Comparison of experimental shear/flexural capacities and model predictions.

No.	$V_{test}$	$V_{M1}$ Er%	$V_{M2}$ Er%	$V_{nh}$ Er%	$V_{h}$ Er%	V Er%	$V_{st}$ Er%
B1	171	9.94%	12.87%	–	–	−43.86%	−15.20%
B2	120	16.67%	15.00%	79.17%	−21.67%	−1.67%	−5.83%
B3	151	31.79%	31.79%	3.97%	−37.75%	6.62%	−3.31%
B4	162	16.05%	19.14%	−3.09%	−41.98%	−40.74%	−10.49%
B5	165	16.97%	18.18%	−4.85%	−43.03%	−29.70%	−10.91%
B6	99	94.95%	96.97%	58.59%	−5.05%	−37.37%	19.19%
B7	161	23.60%	23.60%	−79.50%	−87.58%	−33.54%	−26.71%
B8	102	16.67%	19.61%	141.18%	−7.84%	−5.88%	−1.96%
B9	108	12.96%	14.81%	127.78%	−12.96%	3.70%	−6.48%

**Table 9:** Shear strengths predicted by strut-and-tie model.

No.	$V_{test}$	$V_{M2}$	V	$V_{st}$	$V_{N1}$	$V_{tie}$	$V_{str1}$	$V_{N2}$	$V_{Fc}$	$V_{str2}$
B1	171	193	96	145	178	180	145	356	196	195
B2	120	138	118	113	178	132	113	356	140	149
B3	151	199	161	146	178	182	146	356	185	190
B4	162	193	96	145	178	180	145	356	196	195
B5	165	195	116	147	178	183	147	356	178	186
B6	99	195	62	118	178	183	118	356	178	149
B7	161	199	107	118	178	182	118	356	185	152
B8	102	122	96	100	178	114	100	356	124	134
B9	108	124	112	101	178	116	101	356	113	128

## Data Availability

The original contributions presented in the study are included in the article, further inquiries can be directed to the corresponding author.
